# Synergistic antibacterial activity of silver with antibiotics correlating with the upregulation of the ROS production

**DOI:** 10.1038/s41598-018-29313-w

**Published:** 2018-07-24

**Authors:** Lili Zou, Jun Wang, Yu Gao, Xiaoyuan Ren, Martin E. Rottenberg, Jun Lu, Arne Holmgren

**Affiliations:** 1 Division of Biochemistry, Department of Medical Biochemistry and Biophysics, Karolinska Institutet, SE-171 77 Stockholm, Sweden; 2 Translational Neuroscience & Neural Regeneration and Repair Institute/Institute of Cell Therapy, The People’s Hospital of China Three Gorges University, 443000 Yichang, China; 3 Department of Microbiology, Tumour and Cell Biology, Karolinska Institutet, SE-171 77 Stockholm, Sweden; 4 School of Pharmaceutical Sciences, Southwest University, 400715 Chongqing, China

## Abstract

Thiol-dependent enzymes, including the thioredoxin (Trx) and glutathione (GSH) systems, have recently been found as promising bactericidal targets in multidrug-resistant (MDR) bacteria. We previously discovered that silver acted synergistically with ebselen in the inhibition of the Trx system and also resulted in a fast depletion of GSH in Gram-negative bacteria. Silver has been found by others to improve the sensitivity of bacteria to certain conventional antibiotics. Here, we found that the synergistic antibacterial effects of silver with four conventional antibiotics was correlated with the blockage of bacterial Trx system by silver. The synergistic antibacterial effect came along with the production of reactive oxygen species. All these results suggested that silver primarily enhanced the bactericidal activities of conventional antibiotics towards Gram-negative strains through the upregulation of ROS production.

## Introduction

The worldwide ever-increasing of bacterial resistance to the conventional medical antimicrobial agents is currently one of the most serious health crisis for modern medicine^1–5^. This has serious negative impacts such as decreases of the effectiveness of existing treatments, and causes higher morbidity and mortality rates in patients with infections caused by multidrug-resistant (MDR) bacteria^1,2,6^. European Antimicrobial Resistance Surveillance Network (EARS-Net) latest data showed high and increasing resistance of Gram-negative bacteria, which are combined with resistance to third-generation cephalosporins, fluoroquinolones, and aminoglycosides for both *Escherichia coli* and *Klebsiella pneumoniae*^7^. Given the fact that even education for the public, controlled use in food animals, and prudent antibiotic prescribing^8^ cannot stop the growth and spread of bacterial resistance and can only slow and delay it^2,8,9^. An option to overcome bacterial resistance is the combination of selected antibiotics with each other and with other drugs to improve antibacterial efficacy^1,8,10^.

We and other groups previously found that 2-phenyl-1,2 benzisoselenazol-3(2H)-one (ebselen), is an effective compound against Gram-positive pathogens (including MDR *mycobacteria tuberculosis*) by targeting thioredoxin (Trx), which could transfer electrons from NADPH to their substrates via thioredoxin reductase (TrxR) that is critical for bacterial survival^4,11,12^. Further, the addition of silver acted synergistically with ebselen in treating infections caused by MDR Gram-negative pathogens, including five clinically most difficult-to-treat Gram-negative bacteria: *Acinetobacter baumannii*, *Enterobacter cloacae*, *Escherichia coli*, *Klebsiella pneumonia* and *Pseudomonas aeruginosa*. The combination directly inhibited Trx, deplete glutathione (GSH), and induced striking ROS production^13^. Moreover, the possibility of selection of resistant mutants is very rare^14^. Thus, thiol-dependent antioxidant systems are promising targets for the development of antibiotics against MDR strains.

Dwyer *et al*.^15^ found that antibiotics (ampicillin, gentamicin, and norfloxacin) could alter bacterial redox physiology, and the lethality was accompanied by ROS generation, and Belenky *et al*.^16^ profiled the *E*. *coli* metabolome and showed that antibiotics (ampicillin, kanamycin, and norfloxacin)-induced metabolic alterations elevated redox state, nucleotide oxidation, and increased oxidative stress by tightly regulated glutathione pools. Kohanski *et al*.^17^ stated that all three classes of bactericidal drugs (aminoglycoside, quinolone, and beta-lactam) mediated hydroxyl radical damage. All these above reports pointed out that antibiotics can influence the redox balance in bacteria, but the exact mechanism is still not clear. Meanwhile, Collins and coworkers^17,18^ proved that ROS production is one of the lethal factors of bactericidal antibiotics, and silver can enhance their antibacterial efficacy.

Antibiotic-mediated cell death is a complex, multi-faceted process that cannot be fully accounted for by the direct interactions of antibiotics with specific cellular targets. Here, we try to figure out whether antibiotics representing five different functional categories (beta-lactams, aminoglycosides, synthesis, tetracycline, and macrolides) can work synergistically with silver against Gram-negative bacteria through targeting redox system as ebselen and silver do^13^. Our results showed that silver could enhance certain antibiotics antibacterial effects against Gram-negative bacteria which are correlated to reactive oxygen species (ROS) production.

## Results

### Silver and certain antibiotics in combination exhibited synergistic antibacterial effects against *E*. *coli*

**Figure 1:**
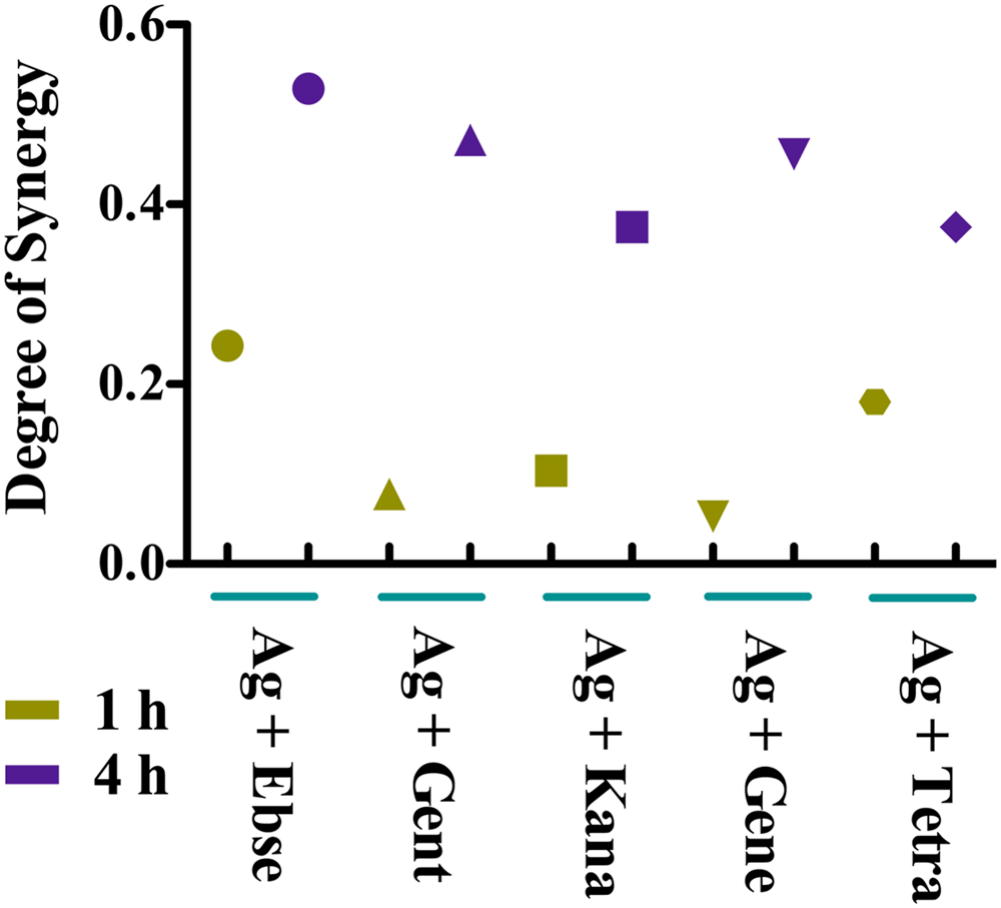
The Bliss model for synergy confirms the synergistic effects, between Ag^+^ and 4 antibiotics, against a model Gram-negative bacteria, *E*. *coli*. The degree of synergy was quantified, using the Bliss Model for Synergy, after 1 and 4 h of treatment with 5 µM AgNO_3_ in combination with the following antibiotics: 80 µM gentamicin, 80 µM kanamycin, 80 µM geneticin, 80 µM tetracycline, and 80 µM Ebselen was used as the positive control.

The antibacterial effects of silver nitrate (AgNO_3_) and nine antibiotics representing five different functional categories (beta-lactams, aminoglycosides, synthesis, tetracycline, and macrolides) on the growth of a model Gram-negative bacterium, *E*. *coli*, which were detected in the 96 wells microplates. Overnight cultured *E*. *coli* DHB4 cells were diluted 1:1000 times in Luria Bertani (LB) medium, and incubated with serial dilutions of ionic silver (Ag^+^) as a nitrate salt and 9 antibiotics in combinations, separately, for 24 h. Ebselen was used as the positive control, which acted synergistically with silver against Gram-negative bacteria in our recent report^13^. The results here showed that 4 (gentamicin, kanamycin, geneticin, tetracycline) out of 9 antibiotics had synergistic activity on *E*. *coli* DHB4 growth under the conditions we tested (Table S1). Further, the Bliss model was used to determine the nature of the therapeutic effects exhibited by the Ag^+^ and antibiotics in combinations. We quantified the degree of synergy at 1 h and 4 h between Ag^+^ and 4 antibiotics (gentamicin, kanamycin, geneticin, and tetracycline) in combinations, and the results showed that Ag^+^ and 4 antibiotics indeed had synergistic antibacterial effects against *E*. *coli* (Fig. 1). All the results pointed out that Ag^+^ could enhance the antibacterial effects of certain antibiotics against Gram-negative bacteria. In the following experiments, we used these 4 antibiotics for further studies.

### ROS was one of the lethal factors for synergistic bactericidal effects of Ag^+^ and antibiotics

**Figure 2:**
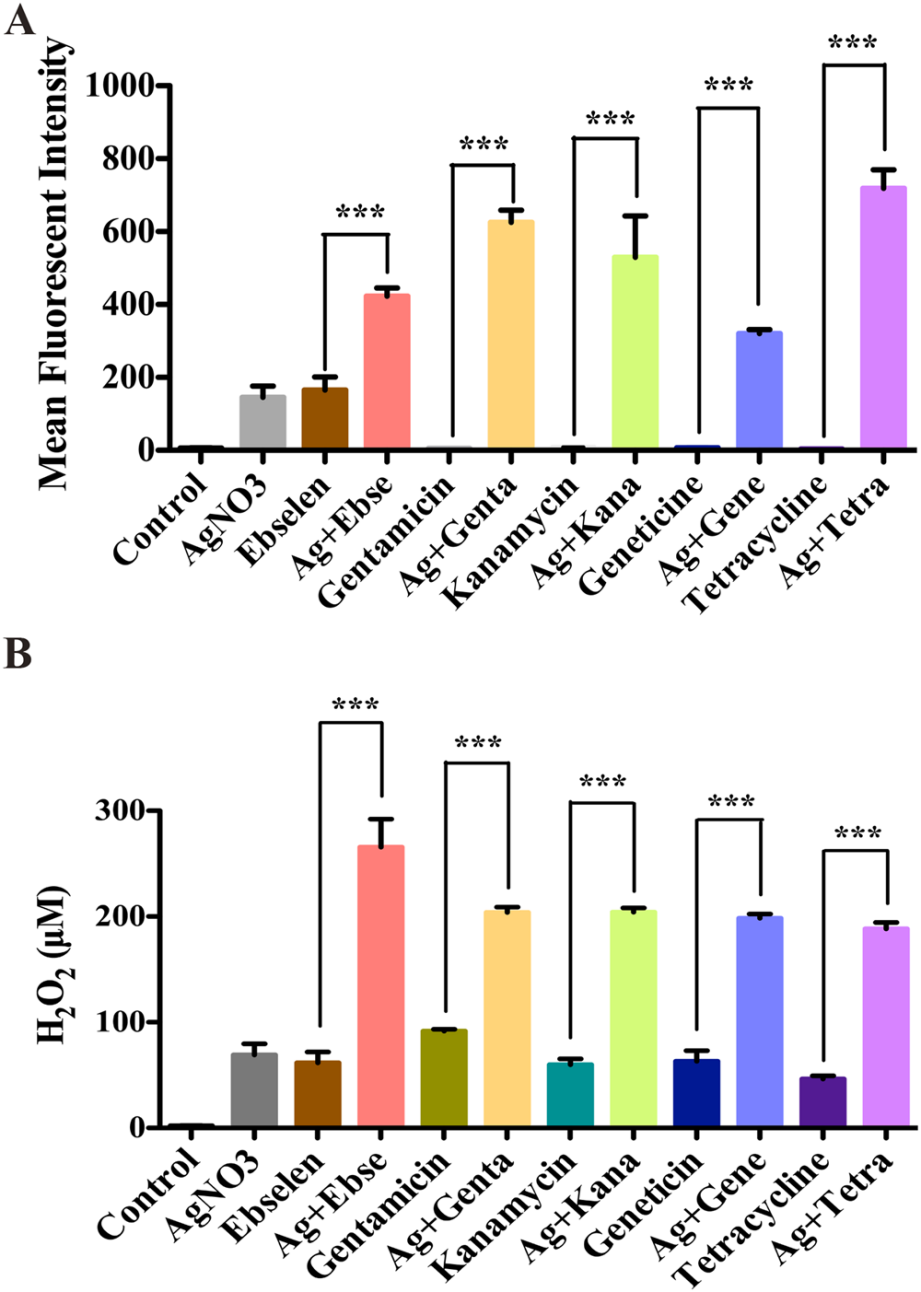
ROS was one of the lethal factors for antibacterial toxicity of silver and antibiotics in combinations. *E*. *coli* DHB4 cells grown to OD_600 nm_ of 0.4 were incubated with 80 µM antibiotics and 5 µM Ag^+^ in combinations, and 80 µM ebselen and 5 µM Ag^+^ in combination was used as the positive control. (**A**) The producion level of ROS was detected by flow cytometry (CyAnadp, Beckman coulter), and mean fluorescent intensity (MFI) ± .s. d. of H_2_DCF-DA-stained *E*. *coli* was detected. (**B**) The production level of H_2_O_2_ was detected by the Amplex® Red Hydrogen Peroxide/Peroxidase method (Invitrogen). Reaction buffer contains 50 μM Amplex® Red reagent, 0.1 U/mL HRP, and the indicated amount of H_2_O_2_ in 50 mM sodium phosphate buffer (pH 7.4), which was incubated for 30 minutes at 25 °C and further verified with absorbance at 560 nm. The background obsorbance was detected by a non-H_2_O_2_ control reaction, which has been subtracted from each value. Data that are presented here as means ± s. d. of 3 independent experiments. *p < 0.05, **p < 0.01, ***p < 0.001 (Student’s *t*-test).

Ag^+^ and ebselen in combination could induce a high level of ROS production^13^, and the effects of Ag^+^ and antibiotics in combinations need further studies. We, therefore, detected the production levels of ROS in Ag^+^ and 4 antibiotics combinations treated cells, and Ag^+^ and ebselen in combination was used as the positive control. The results showed that treatment with 5 μM Ag^+^ alone could not cause a ROS response, and 5 μM Ag^+^ and 80 μM antibiotics in combinations resulted in the upregulation of ROS (*p* < 0.0001) when compare with either antibiotics (Fig. 2A) or silver (Fig. S1A). Furthermore, the increased levels of H_2_O_2_ caused by the treatment with 5 μM Ag^+^ and 80 μM antibiotics in combinations were also notarized by Amplex Red assay^15^ (*p* < 0.0001) (Figs 2B and S1B). All the results demonstrated that ROS might be one of the determining factors for synergistic bactericidal effects of Ag^+^ and antibiotics in combinations against *E*. *coli*.

### Ag^+^ could disrupt bacterial Trx system

**Figure 3:**
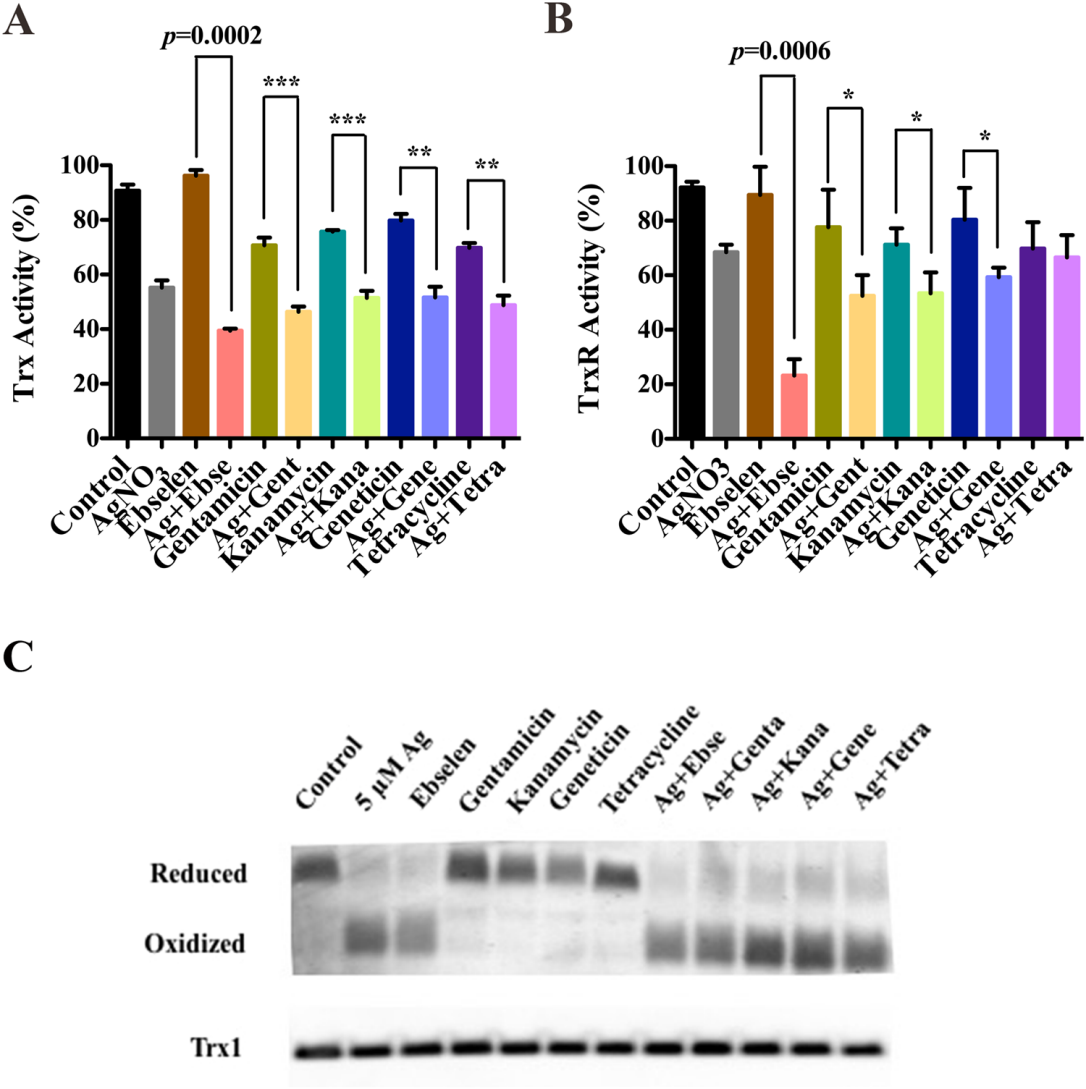
Silver could directly disrupt bacterial Trx system. *E*. *coli* DHB4 cells grown to OD_600nm_ of 0.4 were incubated with antibiotics and Ag^+^ in combinations for 10 min, and ebselen and Ag^+^ in combination was used as the positive control. (**A**) Trx and (**B**) TrxR activities were detected by using DTNB reduction assay in the presence of TrxR or Trx in *E*. *coli* DHB4 cells extracts. (**C**) *E*. *coli* DHB4 cells grown to OD_600 nm_ of 0.4 were incubated with antibiotics and Ag^+^ in combinations for 60 min, and ebselen and Ag^+^ in combination was used as the positive control. *E*. *coli* DHB4 cells extracts were precipitated by 5% TCA, and further alkylated with 15 mM AMS and the redox state of Trx1 was analyzed by redox Western blot. The mean ± s. d. of 3 independent experiments was calculated. The t-test significances were calculated between control and rest groups, and *p < 0.05, **p < 0.01, ***p < 0.001.

One of the main functions of thiol-dependent antioxidant systems is to scavenge ROS to keep cellular redox homeostasis and protect against oxidative stress. The disruption of the Trx and GSH systems may responsible for the upregulation of ROS. Ag^+^ and ebselen in combination has been proven to target both bacterial Trx and GSH systems^13^, while the effects of Ag^+^ and antibiotics in combinations need further studies. *E*. *coli* DHB4 cells grown to OD_600 nm_ of 0.4 were incubated with 5 µM Ag^+^ and 80 µM antibiotics in combinations, and Ag^+^ and ebselen in combination was used as the positive control. Results here showed that after 10 min treatment, the Trx activities in cell extracts treated by Ag^+^ and antibiotics in combinations were dramatically inhibited compared with antibiotics or control group (Fig. 3A, *p* < 0.001); meanwhile, the TrxR activities in cell extracts treated by Ag^+^ and antibiotics in combinations were also statistically lowered when compared with antibiotics or control group (Fig. 3B, *p* < 0.05). We also gained the same results when we prolonged the treatment time to 60 min (Fig. S2). But, there were no statistic difference between Ag^+^ and antibiotics in combinations and silver (Fig. 3, *p* > 0.05). These results suggested that silver could disrupt bacterial Trx system; meanwhile, silver and antibiotics in combinations have direct influences on Trx1 when compared with antibiotics alone.

Bring into correspondence with above observation, the redox state of Trx1 verified by Redox Western Blot was also affected by Ag^+^ and antibiotics in combinations after 60 min treatment when compared to antibiotics. Trx1 was in reduced form in untreated cells (negative control), and became oxidized upon the treatment by Ag^+^ and antibiotics in combinations (Fig. 3C). In contrast, only 10 min treatment by Ag^+^ and antibiotics in combinations could not cause Trx1 oxidization (Fig. S3). At the same time, the total protein levels of Trx1were not affected following a 10 min or 60 min treatment by Ag^+^ and antibiotics in combinations (Figs 3C and S3). These results showed that when targeting Trx system, silver and antibiotics in combinations were not acting as fast as silver and ebselen do, which could effect Trx system in 10 min^13^.

### Ag^+^ and antibiotics could not directly disrupt bacterial GSH system

**Figure 4:**
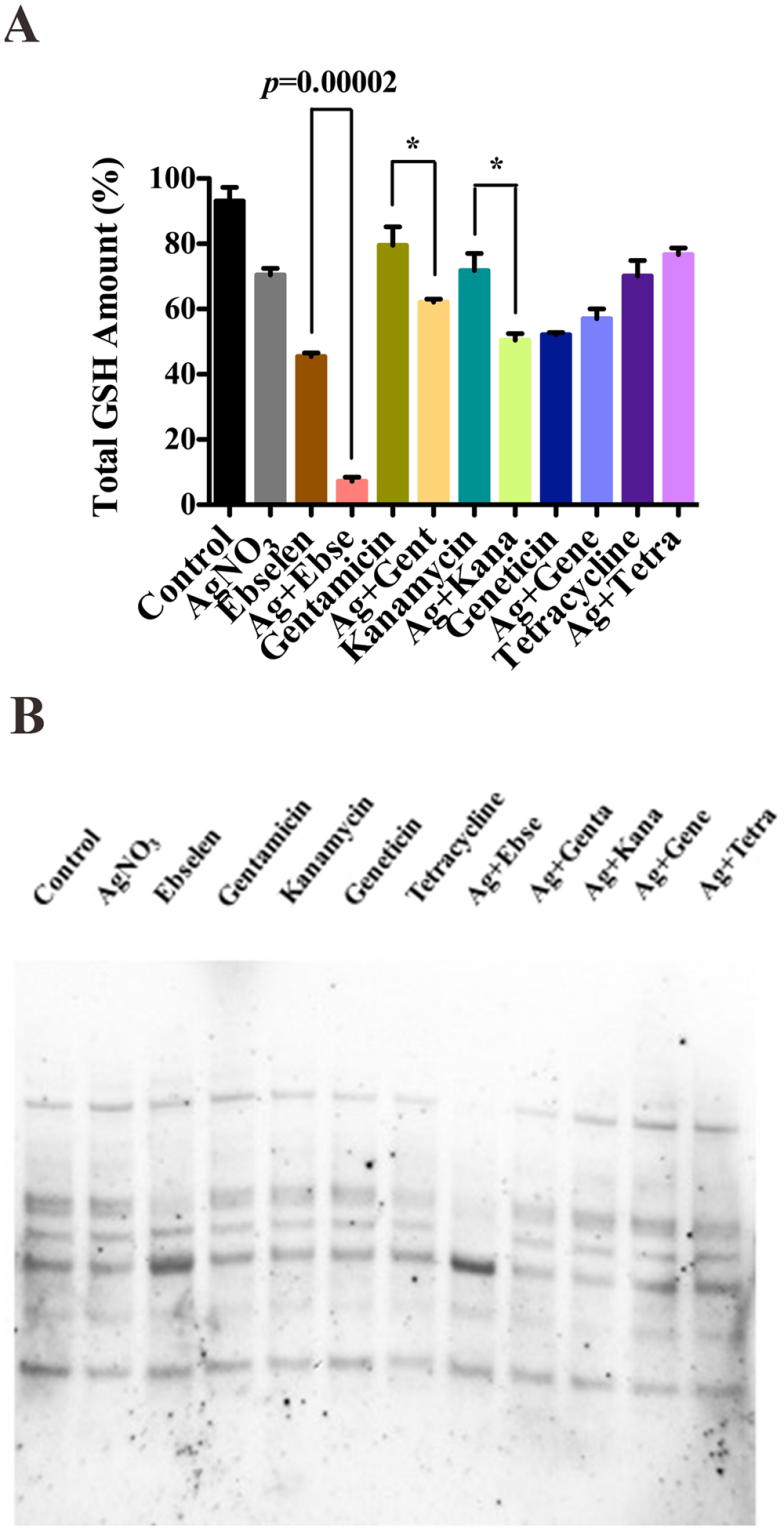
Silver and antibiotics in combinations could not directly disrupt the bacterial GSH system. *E*. *coli* DHB4 cells grown to OD_600nm_ of 0.4 were incubated with antibiotics and Ag^+^ in combinations for 10 min, and ebselen and Ag^+^ in combination was used as a positive control. (**A**) Total amounts of GSH were detected by GR-coupled DTNB reduction assay in *E*. *coli* DHB4 cells extracts. (**B**) Changes in protein *S*-glutathionylation in *E*. *coli*. The mean ± s. d. of 3 independent experiments is calculated. The t-test significances were calculated between control and test groups, and *p < 0.05, **p < 0.01, ***p < 0.001.

**Figure 5:**
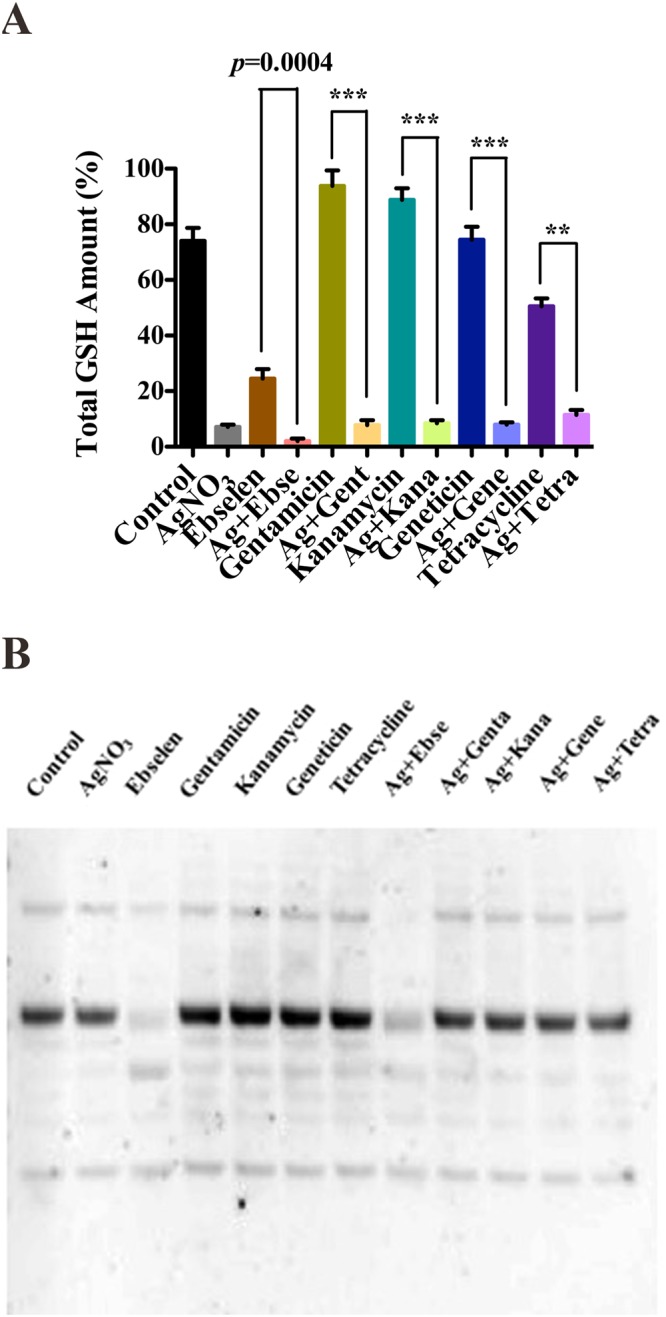
Silver and antibiotics in combinations could not directly disrupt the bacterial GSH system. *E*. *coli* DHB4 cells grown to OD_600 nm_ of 0.4 were incubated with antibiotics and Ag^+^ in combinations for 60 min, and ebselen and Ag^+^ in combination was used as the positive control. (**A**) Total amounts of GSH were detected by GR-coupled DTNB reduction assay in *E*. *coli* DHB4 cells extracts. (**B**) Changes in protein *S*-glutathionylation in *E*. *coli*. The mean ± s. d. of 3 independent experiments was depicted. The t-test significances were calculated between control and rest groups, and *p < 0.05, **p < 0.01, ***p < 0.001.

In our previous work, 5 µM Ag^+^ and 80 μM ebselen in combination has been proven to deplete the GSH after 10 min treatment^13^. In our study, only 5 µM Ag^+^ and 80 μM gentamicin or kanamycin in combinations could slightly deplete the total GSH amount in cell extracts when compared with antibiotics themselves (*p* < 0.05) (Fig. 4A), meanwhile other combinations showed no differences (Fig. 4A) (p > 0.05). Further, the protein *S*-glutathionylation was decreased only in Ag^+^ and ebselen in combination incubated bacteria^13^, but not in those bacteria cells treated with 5 µM Ag^+^ and antibiotics in combinations for 10 min (Fig. 4B) or 60 min treatments (Fig. 5B).

All the results above suggested that silver and antibiotics had no direct effects on bacterial GSH system when acting against Gram-negative bacteria.

### Five most difficult-to-treat MDR Gram-negative clinical isolates were highly sensitive to Ag^+^ and ebselen in combination

We have isolated five clinically most difficult-to-treat MDR Gram-negative pathogen strains: *Acinetobacter baumannii*, *Enterobacter cloacae*, *Escherichia coli*, *Klebsiella pneumonia* and *Pseudomonas aeruginosa*. Overnight cultures of five isolates were diluted 1:1000 times in LB medium, and incubated with serial concentrations of Ag^+^ and antibiotics in combinations for 24 h (Table 1). The results showed that Ag^+^ and antibiotics in combinations exhibited weak antibacterial effects on these MDR Gram-negative bacteria. Meanwhile, Ag^+^ and ebselen in combination might be the only effective antibiotic against a range of resistant bacteria. The results might be explained by the fact that the synergistic antibacterial effect of silver and antibiotics only dependent on the upregulation the ROS production.

**Table 1:** MIC of silver (μM) in the presence of different antibiotics against MDR Gram-negative bacteria.

Antibiotics (4 μM)	MIC of silver (μM)				
	KP-2	AB-1	PA-1	ECL-2	ECO-1
Genta	160	80	40	160	40
Kana	160	80	80	160	40
Gene	160	80	80	160	40
Tetra	160	80	80	160	80
Ebse	40	10	20	10	5

Gentamycin: Genta; Kanamycin: Kana; Geneticin: Gene; Tetracycline: Tetra; Ebse: Ebselen.

## Discussion

Recently a first new antibiotic in decades called Teixobactin was described inhibiting the peptidoglycan cell wall synthesis^3^. However, Teixobactin only inhibits Gram-positive bacteria like *Staphylococcus aureus*. Gram-positive bacteria in general have several differences to Gram-negative one which have a cell wall with an extra membrane. Most of Gram-positive bacteria lack GSH and associated enzymes like glutaredoxins^19^.

The current antibiotics principles are mainly based on disruption of protein synthesis, cell wall or cell membrane assembly, DNA or RNA replication^16^. We recently showed that targeting bacterial thiol-dependent redox enzymes is a distinct antibacterial mechanism compared with traditional antibiotics^4,13^.

There are two prime thiol-dependent enzyme systems, namely, thioredoxin (Trx) system, and glutathione (GSH)/glutaredoxin (Grx) system^19^. For the Trx system, electrons transferoccurs from NADPH to their substrates via thioredoxin reductases (TrxR)^20–23^; meanwhile, for GSH/Grx system, electron transfer from NADPH to their substratesvia glutathione reductases (GR)^21,24,25^. Thiol-dependent enzymes are critical for ribonucleotide reductase (RNR) and DNA replication and repair, ordefense against oxidative stress via peroxiredoxins (Prxs) and methionine sulfoxide reductases (MSRs)^21,24,25^. Therefore, these two systems are critical for cell viability and proliferation.

Trx systems are ubiquitous in all bacteria, whereas the GSH system, as mentioned above, has been found to be absent in many pathogenic bacteria, including nearly all Gram-positive species^19,22,26^. We previously reported that ebselen, which is a substrate of mammalian TrxR but a competitive inhibitor of bacterial TrxR, executs selective antibacterial effect toward Gram-positive bacteria^4,12,27,28^. Further, we recently presented that silver and ebselen in synergistic combination had a strong selective bactericidal effect against Gram-negative bacterial infections. The combination directly inhibited both bacterial Trx and TrxR, and depleted intracellular GSH^13^. Based on these new findings, and combined with other published reports about antibiotics having influence on redox balance^15–17^, and that silver could sensitize Gram-negative bacteria to conventional antibiotics, thus we asked whether they also targeted the redox systems.

Drug-drug interactions can be classified as synergistic, antagonistic, or additive (no interaction). Nine antibiotics used in this work represent five different functional categories (beta-lactams, aminoglycosides, synthesis, tetracycline, and macrolides). Among them, total synthesis and tetracycline are broad-spectrum, aminoglycosides are particularly effective against Gram-negative bacteria, and beta-lactams and macrolides are particularly effective against Gram-positive bacteria. In the work reported here, 4 out of 9 antibiotics acted synergistically with silver against *E*. *coli*, a model Gram-negative bacterium (Fig. 1), which might occur through inducing ROS production as one of the lethal strategies (Figs 2 and 3).

*E*. *coli* has one TrxR, two Trxs (Trx1, and Trx2), and three major thiol peroxidases (bacterioferrit in comigratory protein (BCP), thiol peroxidase (Tpx), and alkyl hydroperoxide peroxidase subunit C (AhpC)^29,30^. Trx1 in *E*. *coli* is involved in protein repair by providing the electrons to *E*. *coli* methionine sulfoxide reductases (Msr)^20^, which participates in the protection of *E*. *coli* against oxidative damage from reactive nitrogen intermediates^31^. Trx1 in *E*. *coli* also acts as a specific reductase for homodimeric Tpx to scavenge ROS^32^. Thus, the decrease of Trx1 activity via its oxidization is at least partially responsible for the ROS production to cause *E*. *coli* cell death. Also the oxidative folding of disulfide containing membrane proteins is dependent on Trx and is possibly targeted^33^.

**Figure 6:**
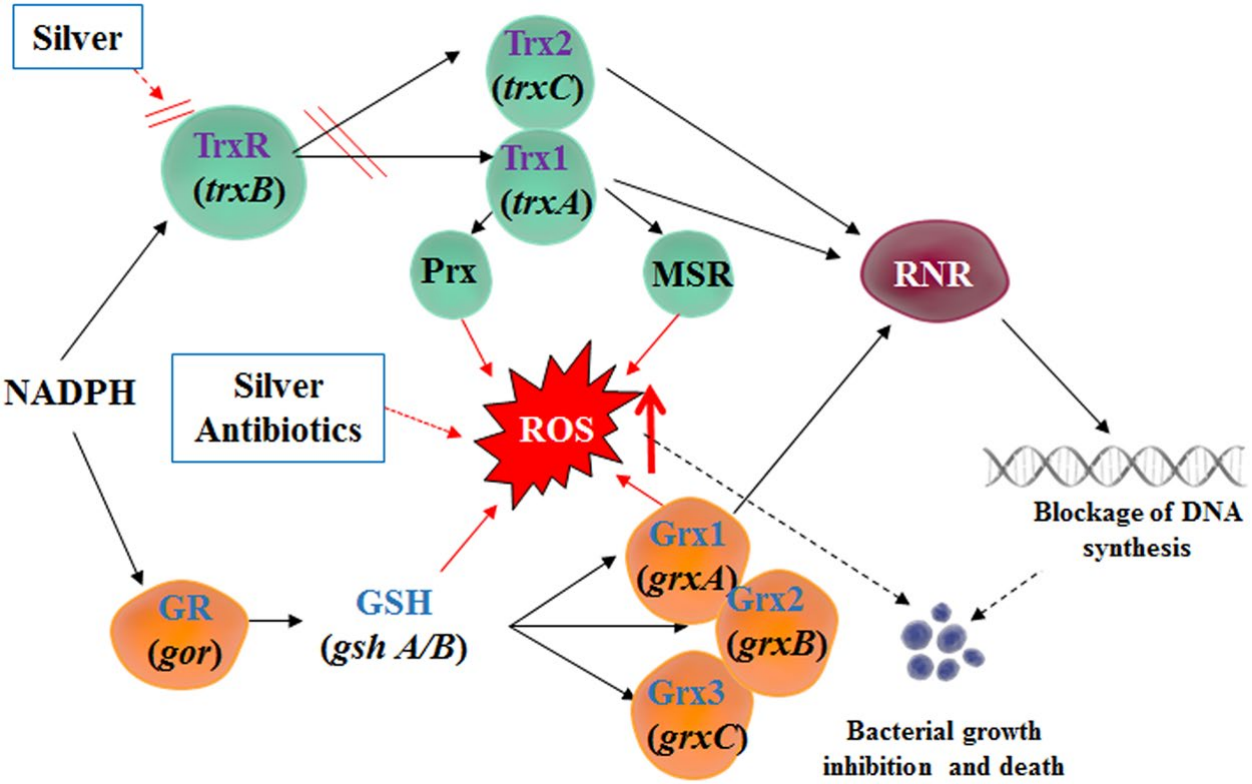
A graphic illustration that showed mechanisms for the synergistic antibacterial activity of Ag^+^ with antibiotics in combination. Antibiotics and Ag^+^ could result in the upregulation of ROS production, and Ag^+^ could directly inhibit *E*. *coli* TrxR and Trx, both of which determine cell death. (**A**) Ag^**+**^ was a strong inhibitor of both *E*. *coli* thioredoxin (Trx) and thioredoxin reductase (TrxR). (**B**) Antibiotics and Ag^**+**^ could result in upregulation of ROS production to determine cell death. (**C**) It is time-dependent. The inhibition is stronger in 60 min treatment than 10 treatment. Information: Trx, thioredoxin; TrxR, thioredoxin reductase; GSH, glutathione; GR, glutathione reductase; Grx: glutaredoxin; Prx, peroxiredoxins; MSR, methionine sulfoxide reductase; RNR, ribonucleotide reductase; ROS, reactive oxygen species.

Meanwhile, although silve could inhibit Trx and TrxR (Fig. 3), yet the effect targeting GSH system is not universal (Figs 4 and 5). The presence of the GSH-Grx system in *E*. *coli* may be regarded as a backup for the Trx system. GSH/Grxs in *E*. *coli* participate in the antioxidant process by deglutathionylation and transfer electrons to ribonucleotide reductase. Silver and antibiotics in combinations showed no effects on GSH amount, and the *S*-glutathionylated proteins are not much different from that of in control group (Figs 4 and 5). Thus, this might be one of the reason to explain the anti-MDR-Gram-negative bacteria activities of silver and antibiotics in combinations were much weaker than silver and ebselen in combination (Table 1 and Fig. 6).

All in all, silver enhanced the antibacterial effects of certain antibiotics against Gram-negative bacteria through upregulate the ROS production. In order to be more effective, the Trx and GSH systems must be targeted, as well, since both systems are particularly important for ribonucleotide reductase in bacteria which is essential for DNA replication and repair.

## Materials and Methods

### Bacterial strains

*Escherichia coli* (*E*. *coli*) DHB4 cells and multidrug-resistance (MDR) Gram-negative clinical isolatess (Tables 2 and 3) were used in *in vitro* experiments. Five Clinical MDR Gram-negative isolates were obtained from clinical patients in People’s Hospital of China Three Gorges University in Hubei Province, PRC, with all approvals and informed consents. All experiments were performed in accordance with relevant guidelines and regulations, and were subject to protocols approved by the Medical Care and Welfare Committee of China Three Gorges University.

**Table 2:** Clinically isolated multidrug-resistant Gram-negative strains used in this work.

Strain	Description	Source
KP-2	*K*. *pneumoniasubsp*. *pneumonia* 0322^#^	This work
AB-1	*Acinetobacter baumannii* (*A*. *baumannii*) H^#^	This work
PA-1	*Pseudomonas aeruginosa* (*P*. *aeruginose*) 1298^#^	This work
ECL-2	*E*. *cloacae* 2301^#^	This work
ECO-1	*Escherichia coli* (*E*. *coli*) 1139^#^	This work

**Table 3:** Drug sensitivity of clinical isolated multidrug-resistant Gram-negative bacteria.

Antibiotic	KP-2	AB-1	PA-1	ECL-2	ECO-1
Amikacin	S	R	S	S	S
Ampicillin	R	R	R/S	/	S
Aztreonam	R	R	R	R	R
Cefazolin	R	R	R	/	R
Cefepime	R	R	R	R	R
Cefotaxime	R	R	R	R	R
Ceftazidime	R	R	R	R	R/S
Chloramphenicol	R	R	/	R	S
Ciprofloxacin	R	R	R	R	R
Gentamicin	R	R	S	R	S
Imipenem	S	R	S	R	S
Levofloxacin	R	R	R	R	R
Meropenem	S	R	R	R	S
Piperacillin	R	R	R/S	R	R
Polymyxin	/	S	S	S	/
Sulbactam	R	R	/	/	R/S
Sulfanilamide	R	/	R	R	R
Tazobactam	R	R	R/S	R	S
Tetracycline	R	R	R	R	R

*R: resistant; S: sensitive.

### Antibiotics and chemicals

Bacteria cells were cultured in Luria Bertani (LB) medium (EMD millipore). 2-Phenyl-1, 2-benzisoselenazol-3(2H)-one (ebselen) (Daiichi), 9 antibiotics (Table 4): ampicillin, carbenicillin, gentamicin, streptomycin, geneticin, kanamycin, chloramphenicol, tetracycline, erythromycin, silver nitrate (Sigma-Aldrich), Methoxypolyethylene glycol maleimide (MeO-PEG-Mal) (Sigma-Aldrich), Iodoacetamide (IAM) (Sigma-Aldrich), protease inhibitor cocktails (Roche), N-acelytcysteine (NAC) (Sigma-Aldrich), DC^TM^ protein assay (Bio-RAD), *E*. *coli* DHB4 TrxR, sheep anti-*E*. *coli* Trx1 antibodywas from IMCO Corp. (Stockholm, Sweden; http://www.imcocorp.se), Rabbit anti-sheep IgG-HRP (Santa cruz), IgG2a mouse monoclonal antibody for glutathione-protein complexes (VIROGEN), 4–12% bolt Bis-Tris gel (VWR), all the other reagents were from Sigma-Aldrich.

**Table 4:** Antibiotics used in the study with their dosages and primary targets.

Antibiotic	Abbreviation	Dose (µM) used	Primary target
Ampicillin	Amp	0/1/2/4	Cell wall formation
Carbenicillin	Car	0/1/2/4	Cell wall formation
Chloramphenicol	Chl	0/1/2/4	Protein synthesis, 50S ribosomal subunit
Erythromycin	Ery	0/1/2/4	Protein synthesis, 50S ribosomal subunit
Gentamicin	Gent	0/1/2/4/80	Protein synthesis, 30S ribosomal subunit
Geneticin	Gene	0/1/2/4/80	Protein synthesis, 30S ribosomal subunit
Kanamycin	Kan	0/1/2/4/80	Protein synthesis, 30S ribosomal subunit
Streptomycin	Str	0/1/2/4	Protein synthesis, 30S ribosomal subunit
Tetracycline	Tet	0/1/2/4/80	Protein synthesis, 30S ribosomal subunit

### Synergistic antibacterial activity of silver and antibiotics in combinations on the *E*. *coli* DHB4 growth

*E*. *coli* DHB4 cells from frozen stock were grown overnight at 37 °C, 400 rpm. The overnight culture was diluted 1:100 with 5 ml of LB medium in 15 ml tubes and incubated at 37 °C at 400 rpm. Overnight cultured DHB4 cells were grown until an OD_600 nm_ of 0.4 and were used for antibiotic treatment. Briefly, DHB4 cells were diluted 1:1,000 into 100 µl of LB medium. Serial dilutions of antibiotics 100 µl (0, 1, 2, 4 µM) and silver nitrate (AgNO_3_, 0, 1.25, 2.5, 5, 10, 20, 40, 80 µM) were added. The minimum inhibitory concentration (MIC) was verified as the lowest concentration of drugs that inhibited 90% of growth compared to the untreated cells after 24 hours culture at 37 °C. The cultures treated with the same serial dilutions of 100 µlebselen and silver nitratewere used as the positive control.

### Quantifying synergy of ebselenand silver using the bliss model

Drug synergism was determined using the Bliss Independence Model, which calculates a degree of synergy using the following formula: S = (f_X0_/f_00_)(f_0Y_/f_00_) − (f_XY_/f_00_), where f_XY_ refers to the wild-type growth rate in the presence of the combined drugs at a concentration X, for one of the drugs, and Y for the other; f_X0_ and f_0Y_ refer to the wild-type growth rates in the presence of the individual drugs at a concentration of X and Y, respectively; f_00_ refers to the wild-type growth rate in the absence of drugs; and S corresponds to the degree of synergy, a parameter that determines a synergistic interaction for positive values and an antagonistic interaction for negative ones. Growth rates at different time points are determined by calculating the slope of the growth or kill curve being analyzed^18^.

### Detection the production of ROS

The *E*. *coli* DHB4 were grown until the OD_600 nm_ of 0.4 in 5 ml LB medium, and the DHB4 cells were incubated with silver and antibiotics in combinations for 10 min. To measure the production level of ROS in the treated bacteria, DHB4 cells were harvested by centrifugation at 6,000 rpm for 5 min and thoroughly washed by PBS, which were further stained by 5 µM H_2_DCF-DA that protected from light for 20 min. After the incubation, treated cells were spun down and re-suspended in PBS, and the amount of ROS was detected by flow cytometry (CyAnadp, Beckman coulter).

### Measurement of H_2_O_2_ Production

The *E*. *coli* DHB4 were grown until the OD_600 nm_ of 0.4 in 5 ml LB medium, and the DHB4 cells were incubated with silver and antibioticsin combinations for 10 min. Treated cells were harvested by centrifugation at 6,000 rpm for 5 min and thoroughly washed 3 times by PBS, and sonicated for 10 s. 50 μl samples were incubated with 50 μM Amplex® Red reagent, and 0.1 U/mL HRP in 50 mM sodium phosphate buffer (pH 7.4) for 30 minutes at 25 °C that protected from light and measured with absorbance at 560 nm (Molecular Probes, Eugene, OR).

### Detection of Trx/TrxR activities and GSH amount in antibiotics and silver treated *E*. *coli* cell lysates

*E*. *coli* DHB4 were grown until the OD_600 nm_ of 0.4 in LB medium, and the DHB4 cells were incubated with 80 µM antibiotics and 5 µM AgNO_3_ for 10 and 60 min, respectively. The cultures that treated with 5 µM AgNO_3_ and 80 µM ebselen were used as the positive control. Treated cells were harvested by centrifugation at 6,000 rpm for 5 min and thoroughly washed 3 times by PBS, and cells were further re-suspended in lysis buffer (100 mM NaCl, 20 mM NaF, 2.5 mM EDTA, 1 mM Na_3_VO_4_, 2.5 mM EGTA, 20 mM sodium ß-glycerophosphate, 0.5% Triton X-100, 10 mM sodium pyrophosphate, 25 mM Tris·HCl (pH 7.5)), which contains protease inhibitor cocktail, and further lysed by sonication. The treated cell lysates were collected by centrifugation at 13,000 rpm for 20 min and the protein concentrations were measured with the Lowry protein kit.

The TrxR activity in *E*. *coli* DHB4 cell extracts was detected by the DTNB reduction activity assay^23^. These experiments were performed in the 96 microwell plates that contains 5 µM *E*. *coli* Trx1, 200 µM NADPH, 1 mM DTNB and 50 mM Tris·HCl (pH 7.5). The absorbance at 412 nm was detected for 5 min with a VERSA microwell plate reader and the slope of initial 2 min was set to verify TrxR activity in cell extracts. The Trx activity was detected by the same method which coupled with 100 nM *E*. *coli* TrxR instead of 5 µM *E*. *coli* Trx in the above discribed reaction solution. To measure the amount of GSH in cell extracts, 25 µg cell lysates were added into the reaction mixture containing 50 nM GR, 200 µM NADPH, 50 mM Tris·HCl (pH 7.5), 1 mM DTNB and 1 mM EDTA. The absorbance at 412 nm was detected for 5 min.

### Redox state of Trx1 in *E*. *coli* treated by silver and antibiotics in combinations

*E*. *coli* DHB4 were grown until the OD_600 nm_ of 0.4 in LB medium, and the DHB4 cells were incubated with 80 µM antibiotics and 5 µM AgNO_3_ for 10 and 60 min, respectively. The cultures treated with 5 µM AgNO_3_ and 80 µM ebselen were used as the positive control. Redox Western blotting was performed to detect the redox state of Trx1 in the DHB4 cells. The treated cells werefurther harvested by centrifugation at 6,000 rpm for 5 min and thoroughly washed 3 times by PBS, and the protein was precipitated by 5% TCA in 1.0 ml. The precipitates were washed throughly 3 times with 1 ml pre-ice-cold acetone and dissolved in 50 mM Tris·HCl (pH 8.5) with 0.5% SDS containing 15 mM MeO-PEG-Mal at 37 °C for 2 hours. Proteins were collected by centrifugation at 13,000 rpm for 20 min and the protein concentration was verified with the Lowry protein kit. Proteins were incubated with SDS-loading buffer at 90 °C for 10 min, and then separated on the 4–12% bolt Bis-Tris gel with MES running buffer (150 V, 40 min). The redox state of Trx1was detected withsheep anti-*E*. *coli* Trx1 antibody at 1:1000 dilution, followed by the detection of Chemiluminescence Reagent Plus.

### Proteins *S*-glutathionylation in *E*. *coli* cells treated by silver and antibiotics in combinations

Total protein *S*-glutathionylation of antibiotics and AgNO_3_ in combinations treated *E*. *coli* DHB4 cells was verified by Western blot. *E*. *coli* DHB4 were grown until the OD_600 nm_ of 0.4 in LB medium, and the DHB4 cells were incubated with 80 µM antibiotics and 5 µM AgNO_3_ for 10 and 60 min, respectively. The cells treated with 5 µM AgNO_3_ and 80 µM ebselen were used as the positive control. Cells were washed 3 times, and re-suspended in lysis buffer (10 mM sodium pyrophosphate, 100 mM NaCl, 2.5 mM EDTA, 25 mM Tris·HCl (pH 7.5), 2.5 mM EGTA, 1 mM Na_3_VO_4_, 20 mM sodium ß-glycerophosphate, 0.5% Triton X-100, 20 mM NaF and 50 mM IAM) containing protease inhibitor cocktail. Afte sonication, the treated cell lysates were collected by centrifugation at 13,000 rpm for 20 min. Protein concentration was measured with Lowry protein kit, and Western blot was performed as described above with IgG2a mouse monoclonal antibody (VIROGEN, 101-A/D8) for *S*-glutathione-protein complexes.

### Antibacterial activities of antibiotics and silver on the growth of MDR Gram-negative clinical isolates

Five MDR Gram-negative clinical isolates were grown till an OD_600 nm_ of 0.4, and were diluted 1:1,000 into 100 µl LB medium. Serial dilutions of 100 µl antibiotics (0, 1, 2, 4 µM) and AgNO_3_ (0, 1.25, 2.5, 5, 10, 20, 40, 80, 160 µM) in combinations were added to the individual wells. The MBC was verified after 16 hours culture at 37 °C. The cells treated with the same serial dilutions of 100 µl ebselen and silver nitratewere used as the positive control.

### Statistical analysis

Mean, Standard Deviation (SD) and t-test (two tails, unpaired) significances were calculated in GrapPad Prism Software. *p < 0.05, **p < 0.01, ***p < 0.001.

### Data availability

The data sets generated during and/or analyzed during the current study are available from the corresponding author on reasonable request.

## Electronic supplementary material

supplementary figures

supplementary information
