# Supplementary material for: Synergistic antibacterial activity of silver with antibiotics correlating with the upregulation of the ROS production
Source: Sci Rep. 2018 Jul 24;8:11131. doi: 10.1038/s41598-018-29313-w (PMC6057937; doi:10.1038/s41598-018-29313-w)
Supplement: Supplementary file 1 — supplementary figures [file 41598_2018_29313_MOESM1_ESM.pdf]

Synergistic antibacterial activity of silver with antibiotics correlating with the upregulation of the ROS production

Running Title: silver antibacterial activity and the increase of ROS production

Authors: Lili Zou[1,2,#], Jun Wang[2,#], Yu Gao[3], Xiaoyuan Ren[1], Martin E. Rottenberg[3], Jun Lu[1,4,*], Arne Holmgren[1,*]

Authors Affiliations:

[1]Division of Biochemistry, Department of Medical Biochemistry and Biophysics, Karolinska Institutet, SE-171 77, Stockholm, Sweden.

[2]Translational Neuroscience & Neural Regeneration and Repair Institute/Institute of Cell Therapy, The People's Hospital of China Three Gorges University, 443000 Yichang, China.

[3]Department of Microbiology, Tumour and Cell Biology, Karolinska Institutet, SE-171 77 Stockholm, Sweden.

[4]School of Pharmaceutical Sciences, Southwest University, 400715 Chongqing, China.

*Correspondence to: arne.holmgren@ki.se, Tel.: 46-70-6467686, Fax: 46-8-7284716; and junlu@swu.edu.cn.

[#]These authors equally contributed to this work.

Supplementary figures

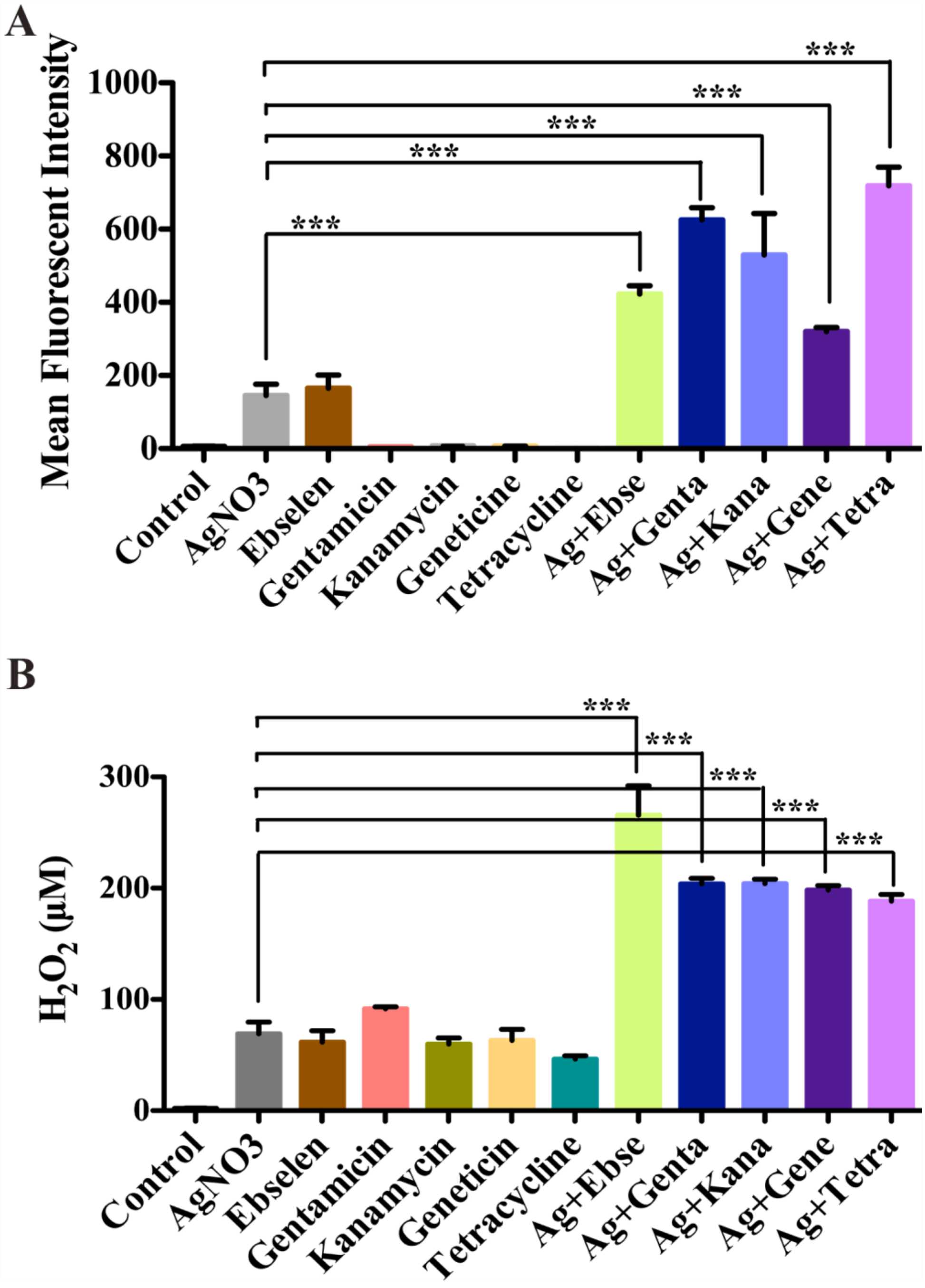

Figure S1. ROS was one of the key factors for synergistic bactericidal effects of silver and antibiotics in combinations. *E. coli* DHB4 grown to OD_{600nm} of 0.4 were treated with 80 μM antibiotics and 5 μM $AgNO_3$ in combinations, and silver and ebselen in combination was used as a positive control. **A.** ROS level was detected by flow cytometry (CyAnadp, Beckman coulter), and mean fluorescent intensity (MFI) ±. s. d.of H_2DCF-DA-stained *E. coli* were detected. **B.** Detection of H_2O_2 using the Amplex® Red Hydrogen Peroxide/Peroxidase Assay Kit (Invitrogen). Data are presented as means ± s. d. of three independent experiments. *: p<0.05, **: p< 0.01, ***: p<0.001 (Student's t-test).

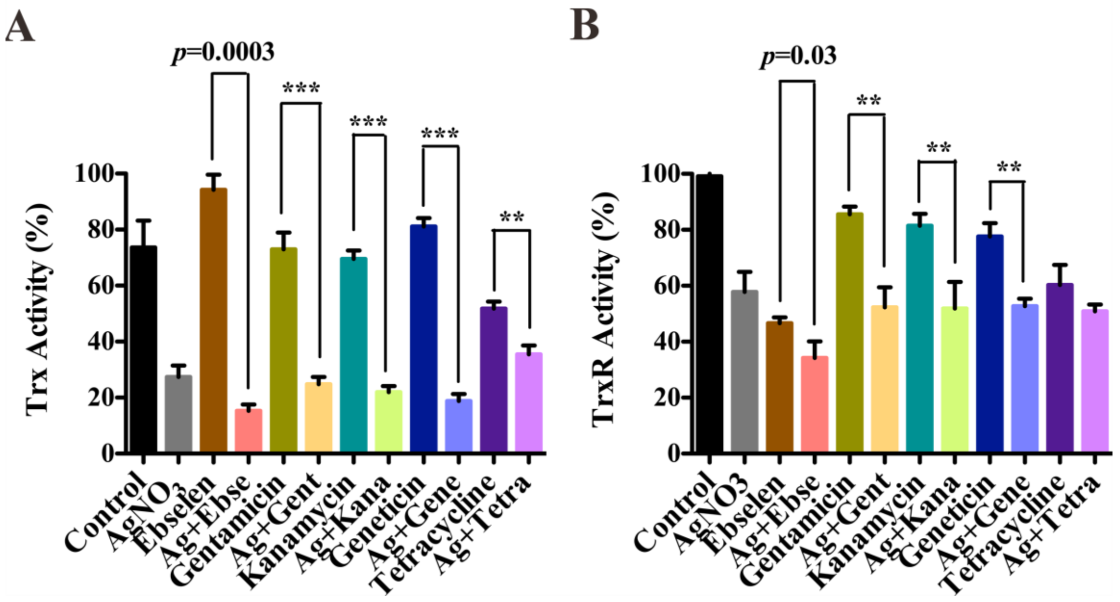

Figure S2. Silver and antibiotics in combinations could directly disrupt bacterial Trx system. *E. coli* DHB4 grown to OD_{600nm} of 0.4 were treated with antibiotics and $AgNO_3$ in combinations for 60 min, and ebselen and $AgNO_3$ in combination was used as positive a control. **A.** Trx and **B.** TrxR activities were assayed using DTNB reduction in the presence of TrxR or Trx in *E. coli* extracts. The mean ± s. d. of three independent experiments was depicted. The t-test significances were calculated between control and rest groups, and *: $p<0.05$, **: $p< 0.01$, ***: $p<0.001$.

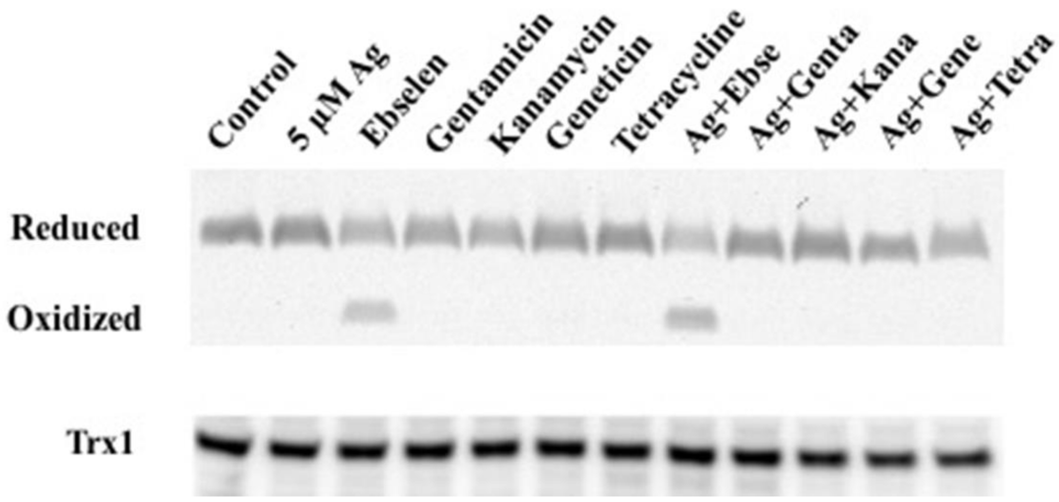

Figure S3. Silver and antibiotics in combinations could not rapidly disrupt bacterial Trx system. *E. coli* DHB4 grown to OD_{600nm} of 0.4 were treated with antibiotics and $AgNO_3$ in combinations for 10 min, and ebselen and $AgNO_3$ in combination was used as positive control. *E. coli* were precipitated in 5% TCA and alkylated with 15 mM AMS and the degree of reduced Trx1 was analyzed by Western blot.
