# Supplementary material for: Synergistic antibacterial activity of silver with antibiotics correlating with the upregulation of the ROS production
Source: Sci Rep. 2018 Jul 24;8:11131. doi: 10.1038/s41598-018-29313-w (PMC6057937; doi:10.1038/s41598-018-29313-w)
Supplement: Supplementary file 2 — supplementary information [file 41598_2018_29313_MOESM2_ESM.docx]

**Table S1 MIC of silver (μM) in combination with nine antibiotics against wild type *E. coli* DHB4**

| **Antibiotics**  **Concentration**  **(μM)** | **MIC of silver (μM)** | | | | | | | | | |
| --- | --- | --- | --- | --- | --- | --- | --- | --- | --- | --- |
|  | **Beta-lactams** | | **Aminoglycosides** | | | | **Tetracycline** | **Macrolides** | **Synthesis** | **Control** |
|  | **Amp** | **Car** | **Genta** | **Strep** | **Gene** | **Kana** | **Tetra** | **Ery** | **Chlor** | **Ebse** |
| **0** | 40 | 40 | 40 | / | 40 | 40 | 40 | 40 | 40 | 40 |
| **1** | 40 | 40 | 20 | / | 40 | 20 | 20 | 40 | 40 | 10 |
| **2** | 40 | 40 | 2.5 | / | 10 | 2.5 | 10 | 40 | 40 | 5 |
| **4** | 40 | 40 | 1.25 | 40 | 2.5 | 1.25 | 1.25 | 40 | 40 | 1.25 |

Ampiciline: Amp; Carbenicillin: Car; Gentamycin: Genta; Streptomycin: Strep; Geneticin: Gene; Kanamycin: Kana; Tetracycline: Tetra; Erythromycin: Ery; Chloramphenicol: Chlo; Ebse: Ebselen. Streptomycin: 8 μM Streptomycin and 40 μM silver in combination can kill *E. coli* DHB4, which showed no synergistic effect.
